# Cognitive impairment in chronic respiratory disease patients using long-term oxygen therapy: a narrative review

**DOI:** 10.4103/mgr.MEDGASRES-D-25-00116

**Published:** 2026-01-06

**Authors:** Hiroki Annaka

**Affiliations:** Department of Occupational Therapy, Faculty of Rehabilitation, Niigata University of Health and Welfare, Niigata, Japan

**Keywords:** cannula tubes, healthcare, hypoxemia, hypoxia, inflammation, lung function, medication, oxygen, pulmonary disease, smoking

## Abstract

Long-term oxygen therapy is used to treat of chronic respiratory diseases with chronic hypoxia. To date, long-term oxygen therapy has significantly contributed to the relief of dyspnea in the daily life of patients with chronic respiratory disease and chronic hypoxemia. Chronic hypoxia is a possible cause of cognitive impairment, and patients with chronic respiratory disease using long-term oxygen therapy with severe chronic hypoxia may be at a higher risk of cognitive impairment than patients using non-long-term oxygen therapy. Cognitive impairment in patients with chronic respiratory disease can lead to a decline in treatment adherence, including medication usage, health care check-ups, and smoking cessation efforts, which contribute to disease progression. In addition, patients using long-term oxygen therapy require oxygen delivery equipment. Operating oxygen delivery equipment is difficult for patients with cognitive impairment, and the inability to use long-term oxygen therapy properly is a serious challenge that can affect their life expectancy. Patients with chronic respiratory disease who use long-term oxygen therapy may be more affected by cognitive impairment than non-long-term oxygen therapy patients. Several review articles have addressed cognitive impairment in patients with chronic respiratory disease; however, none specifically focus on patients with chronic respiratory disease using long-term oxygen therapy. This narrative review describes the current knowledge and future issues regarding cognitive impairment in patients with chronic respiratory disease using the long-term oxygen therapy.

## Introduction

Long-term oxygen therapy (LTOT) involves the treatment of patients with chronic hypoxemia who require oxygen inhalation for at least 15 hours per day. This treatment was first reported in 1967,^1^ and two RCTs in the 1980s demonstrated its efficacy in prolonging the survival of patients with chronic respiratory disease (CRD) and hypoxemia.^2^,^3^ The development of oxygen delivery equipment facilitates the continuation of oxygen inhalation at home and promotes its widespread use of this therapy.^4^,^5^ To date, LTOT has significantly contributed to the relief of dyspnea in the daily life of patients with CRD and chronic hypoxemia.

Cognitive impairment is widely known to be a common comorbidity among patients with CRD. Cognitive impairment may be caused by multiple factors, which may vary among each patient.^6^,^7^ Among these, chronic hypoxemia is the most challenging.^8^,^9^ Recent studies have elucidated the mechanisms through which hypoxia leads to neuronal damage.^10^,^11^,^12^ Neuronal damage induced by hypoxia is thought to involve three key mechanisms: (1) inhibition of synaptophysin synthesis, (2) induction of inflammation and oxidative stress via the release of proinflammatory cytokines and reactive oxygen species, and (3) endothelial dysfunction and impaired cerebral blood flow due to activation of endothelial nitric oxide synthase.^10^,^11^,^12^ Furthermore, hypoxemia is associated with a decline in lung function, including unfavorable changes in parameters such as forced expiratory volume in 1 second, forced vital capacity, and diffusing capacity for carbon monoxide, and a decrease in oxygen supply to the brain.^8^,^9^ This condition poses a threat to the brain due to the physiological stress caused by inflammatory mediators.^13^ As a result, it leads to cognitive impairment due to organic brain damage.^14^ Decreased lung function and severe hypoxemia are associated with a higher risk of dementia and severe cognitive impairment. This hypothesis is supported by large cohort studies^15^,^16^ and meta-analyses.^14^,^17^ The most important concern regarding cognitive impairment is its negative impact on the treatment of patients with CRD.^18^ It may reduce adherence to treatment and contribute to disease progression.^18^,^19^ Therefore, cognitive impairment in CRD is a critical factor to consider when planning a patient’s treatment plan.^20^,^21^

Patients with CRD using LTOT have exhibited specific characteristics compared to non-LTOT patients. One example is severe hypoxia in the absence of oxygen inhalation.^5^,^22^ Another is the lifestyle adjustment required to coexist with oxygen delivery equipment and cannula tubes.^23^,^24^,^25^ These unique characteristics of CRD patients using LTOT, such as severity and lifestyle, may differ from those of non-LTOT patients in terms of the negative effects of cognitive impairment.

Unfortunately, while a few review articles address cognitive impairment in CRD patients generally, no review articles to date have focused specifically on CRD patients using LTOT. Furthermore, many existing review articles primarily examine the mechanisms underlying cognitive impairment in patients with CRD, such as hypoxemia, while providing limited insight into the specific characteristics of cognitive impairment in those using LTOT and its potential impact on patient’s outcomes and prognosis. Therefore, this narrative review summarizes current knowledge on cognitive impairment in patients with CRD using LTOT and offers directions for future research. Particular emphasis is placed on the effects of cognitive impairment on daily functioning and long-term prognosis in patients with CRD using LTOT.

## Search Strategy

This study was searched using PubMed. The search used the following formula: “long-term oxygen therapy” AND “cognitive” AND “chronic respiratory disease.” All years were included in the search.

## Cognitive Impairment in Chronic Respiratory Disease Patients using Long-Term Oxygen Therapy

According to the global strategy for prevention, diagnosis and management of COPD: 2025 report, the prevalence of cognitive impairment in chronic obstructive pulmonary disease (COPD) is approximately 36% or 56%, depending on cognitive function tests.^26^ This percentage includes patients with CRDs of all severities, including those without chronic hypoxia and those using LTOT.^27^,^28^ Our study, limited to patients with CRD using LTOT, showed a 70% prevalence of cognitive impairment, as measured by the Montreal Cognitive Assessment.^29^ Our results suggest that patients with CRD using LTOT may be at an increased risk of comorbid cognitive impairment. However, this cross-sectional study comprised data from a single center, which limits the generalizability of the results. Large-scale studies are warranted to investigate the prevalence of cognitive impairment in CRD patients using the LTOT.

**Table 1** presents a summary of previous studies evaluating cognitive function in patients with CRD using LTOT. Several studies have compared the cognitive functions of patients with CRD using LTOT and those with CRD using non-LTOT. Mermit Çilingir et al.^30^ compared patients with acute exacerbation of COPD using LTOT to those of non-LTOT on the Mini-Mental State Examination and indicated significantly lower scores for those using LTOT. In a study of patients with idiopathic pulmonary fibrosis by Bors et al.,^31^ the severe idiopathic pulmonary fibrosis group using the LTOT performed poorly on the Trail Making Test, Stroop Test, Hopkins Language Learning Test, and Boston Naming Test compared to the mild-to-moderate group, including some patients using the LTOT. These two studies showed that patients using LTOT had a more severe decline in lung function, as measured by forced expiratory volume in 1 second, forced vital capacity, and diffusing capacity for carbon monoxide, than those who did not use LTOT. Karamanli et al.^32^ compared cognitive function, measured by the Montreal Cognitive Assessment and Mini-Mental State Examination, in patients with COPD using LTOT and those who did not use LTOT with comparable lung function and found that patients using LTOT performed better. Similarly, in a study by Dal Negro et al.,^33^ patients with COPD using LTOT showed better performance in Trail Making Test than COPD patients not using LTOT when they had comparable lung function. These results suggest that the cognitive function of patients with CRD using LTOT due to severe lung function decline is lower than that of non-LTOT patients with mild lung function decline but higher than that of non-LTOT patients with comparable lung function. One concern is whether LTOT is effective in preventing cognitive decline. In our one-year prospective cohort study of patients with CRD using LTOT, we observed progressive cognitive decline in 40% of the patients.^34^ In addition, Ohrui et al.^35^ reported similar results; however, interestingly, this study showed that cognitive decline progressed faster in women than in men. In other words, LTOT has the potential to slow the progression of cognitive decline in patients with CRD and chronic hypoxia but may not be sufficient to fully preserve cognitive function. Routine cognitive testing using LTOT may be required for patients with CRD to account for their progressive cognitive decline. This can help detect the early signs of cognitive decline and establish a care plan for patients with cognitive impairment.

**Table 1:** Summary of studies assessing cognitive function in patients with chronic respiratory disease using long-term oxygen therapy

Design	Subject	Control	Outcome	Results	Reference
Cross-sectional study	Chronic respiratory disease on long-term oxygen therapy (*n* = 96)	None	Montreal Cognitive Assessment	Sixty-seven patients (70%) had a Montreal Cognitive Assessment score of less than 24 points.	Annaka et al.^29^
Case-control study	Regular user long-term oxygen therapy dependent-COPD (n = 22)	Nonuser long-term oxygen therapy dependent-COPD (*n* = 62)	Mini-Mental State Examination	Mini-Mental State Examination scores for regular long-term oxygen therapy dependent-COPD were lower than those for non-regular long-term oxygen therapy dependent-COPD (18.81 ± 3.65 scores, 24.9 ± 5.17 scores, *P* < 0.001).	Mermit Çilingir et al.^30^
Case-control study	Severe idiopathic pulmonary fibrosis (*n* = 12; Home O_2_ user = 12)	Mild to moderate idiopathic pulmonary fibrosis (*n* = 34. Home O_2_ user = 21) Control (*n* = 15)	Trail Making Test, Stroop Color Word Test, Hopkins Verbal Learning Test, Boston Naming Test	Severe idiopathic pulmonary fibrosis patients had poorer cognitive function than mild idiopathic pulmonary fibrosis patients or controls (Trail Maling Test-A: mean 42.3 ± 12.9 s, mean 33.5 ± 10.0 s, 33.1 ± 9.8 s, *P* = 0.04; Trail Maling Test-B: mean 135.9 ± 69.4 s, mean 86.7 ± 34.9 s, 83.2 ± 35.4 s, *P* < 0.01; Stroop Color Word Test 2: 49 ± 18.8, 59 ± 11.0, 63 ± 10.1, *P* = 0.02; Stroop Color Word Test 3: 20 ± 11.7, 30 ± 9.3, 38 ± 10.1, *P* < 0.01; Hopkins Verbal Learning Test: 7.7 ± 2.4, 7.8 ± 2.3, 9.7 ± 1.7, *P* = 0.01; Boston Naming Test: 52.5 ± 5.4, 55.4 ± 3.3, 56.7 ± 2.5, *P* = 0.01).	Bors et al.^31^
Case-control study	Regular-user long-term oxygen therapy dependent-COPD (*n* = 21)	Nonuser long-term oxygen therapy dependent-COPD (*n* = 24)	Mini-Mental State Examination, Montreal Cognitive Assessment	Mini-Mental State Examination and Montreal Cognitive Assessment were higher in regular long-term oxygen therapy dependent-COPD than in nonuser long-term oxygen therapy dependent-COPD (Mini-Mental State Examination: *P* = 0.014; Montreal Cognitive Assessment: *P* = 0.007).	Karamanli et al.^32^
Case-control study	COPD regularly used long-term oxygen therapy (*n* = 73)	COPD only as needed long-term oxygen therapy (*n* = 73)	Mini-Mental Status test, Clock Drawing test, Trial Making test	COPD regularly used long-term oxygen therapy performed better on the Trail Making Test than COPD only as needed long-term oxygen therapy (Trail Making Test A: 132.2 ± 35.8 s, 155.3 ± 52.5 s, *P* = 0.012; Trail Making Test B: 332.1 ± 36.2 s, 344.2 ± 31.8 s, *P* = 0.001).	Dal Negro et al.^33^
Prospective observational study	Chronic respiratory disease undergoing long-term oxygen therapy (*n* = 55)	None	Montreal Cognitive Assessment	Twenty-two patients (40%) showed cognitive decline as defined by the minimal clinically important difference in the Montreal Cognitive Assessment.	Annaka et al.^34^
Cross-Sectional study	Chronic respiratory disease with long-term domiciliary oxygen therapy (*n* = 135)	Chronic respiratory disease with non-long-term domiciliary oxygen therapy (*n* = 718)	Mini-Mental State Examination	Mini-Mental State Examination scores showed that age-related cognitive decline was more pronounced in female patients than in female controls (-0.524/year, *R^2^* = 0.426, -0.120/year, *R^2^* = 0.027, *P* < 0.0001).	Ohrui et al.^35^

COPD: Chronic obstructive pulmonary disease.

However, most studies examining cognitive impairment in patients with CRD using LTOT should be interpreted with caution, as they often rely on small sample sizes from single-centers or non-randomized case-control designs.

## Effects of Cognitive Impairment in Chronic Respiratory Disease Patients using Long-Term Oxygen Therapy

The effects of cognitive impairment on CRD patients include a decline in medication adherence,^36^ difficulty in operating inhalers,^18^ a decline in adherence to health care,^37^ and decreased smoking cessation rates.^38^ These effects cause frequent exacerbations resulting in disease progression.

Unlike non-LTOT patients, CRD patients using LTOT are required to operate oxygen delivery equipment in their daily lives. Adherence to LTOT remains suboptimal. A review by Katsenos et al.,^39^ reported that 30–55% of patients failed to adhere to prescribed LTOT regimes. Low adherence has been attributed, in part, to the complexity of operating oxygen supply equipment, which involves multiple procedural steps and may be particularly challenging for CRD patients with cognitive impairment.^39^,^40^,^41^ Specific processes that are made difficult by cognitive impairment include (1) keeping the oxygen supply equipment away from fire, (2) setting a predetermined oxygen flow, (3) switching to an external cylinder or equipment, (4) handling the cannula tube and oxygen supply equipment, (5) responding to alarm activation, and (6) difficulty in fitting the cannula tube.^40^,^42^,^43^ Gauthier et al.^41^ identified age as a predictor of adherence to LTOT. In our previous study, we evaluated the ability of patients with normal cognitive function and those with mild cognitive impairment to self-manage the operation of oxygen delivery equipment.^40^ The results revealed that patients with mild cognitive impairment demonstrated significantly lower self-management ability for oxygen supply equipment compared to those with normal cognitive function. Furthermore, lower scores on the Montreal Cognitive Assessment were associated with a reduced ability to independently operate the equipment.^40^ This can result in fires, explosions, falls, and inadequate oxygen inhalation.^40^,^42^,^43^ Cognitive impairment is also associated with increased sedentary time in CRD patients using LTOT.^29^ This may be due to the patient’s inability to successfully handle an extended oxygen tube for inhalation at home or external oxygen supply equipment, which limits the patient’s range of activities.^25^,^29^ Approximately 75% of patients experienced falls associated with oxygen delivery equipment, and fear of falling may contribute to increased sedentary behavior.^29^,^43^

Thus, the effects of cognitive impairment in CRD patients using LTOT may not only reduce adherence to treatment, as observed in CRD patients with non-LTOT, but may also render oxygen supply difficult. However, research exploring the relationship between cognitive impairment and the ability to self-manage LTOT in patients with CRD remains extremely limited. Moreover, existing studies are primarily small-sample cross-sectional investigations, highlighting the need for further in-depth research in this area.

## Effects of Cognitive Impairment on Mortality in Patients with Chronic Respiratory Disease using Long-Term Oxygen Therapy

Older adults with mild cognitive impairment have higher mortality rates when CRD is a comorbid condition compared to healthy older adults.^44^ This has been shown to accelerate disease progression with exacerbations due to a decline in treatment adherence.^44^ Although studies on cardiovascular disease have provided evidence for the prognostic value of a decline in treatment adherence due to cognitive impairment, to the best of our knowledge, no studies have been conducted examining CRD in this context.^45^,^46^

**Figure 1:**
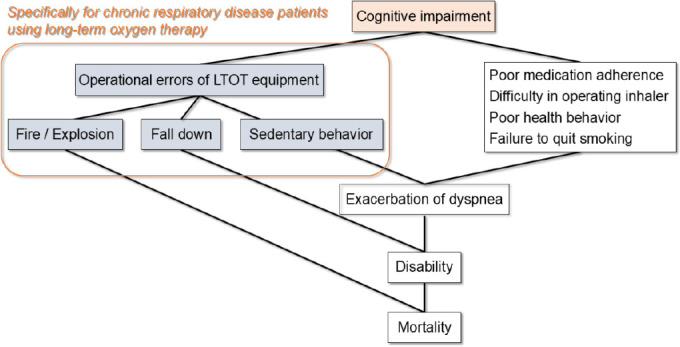
Effects of cognitive impairment in patients with chronic respiratory disease using long-term oxygen therapy (LTOT). This figure shows the effect of cognitive impairment in chronic respiratory disease patients using LTOT and its relationship to mortality. The colored boxes show the effect specific to patients with chronic respiratory disease using LTOT that is not present in patients who do not use LTOT. Created with Microsoft PowerPoint.

Our previous study showed that cognitive function may predict 2-year mortality in patients with CRD using LTOT.^47^ The aforementioned challenges with the operation of oxygen supply equipment due to cognitive impairment are presumed to be factors affecting the life expectancy of patients with CRD using LTOT. Large-scale cohort studies have shown that LTOT use reduces the incidence of acute exacerbations and hospital readmission.^48^ These findings suggest that adherence to LTOT plays a beneficial role in slowing disease progression. Fires and explosions caused by placing the oxygen supply equipment near a fire can lead to accidental deaths.^49^ Falls due to contact with cannula tubes, oxygen delivery equipment, and inadequate oxygen inhalation lead to increased dyspnea and frequent hospitalizations in patients with CRD using LTOT, gradually leading to activity limitations and disease progression.^50^,^51^ Based on these considerations, cognitive impairment is not directly related to mortality but may be a factor that, in combination with other factors, accelerates the progression of CRD in patients using LTOT.^47^,^48^,^49^ **Figure 1** shows the relationship between cognitive impairment and mortality in patients with CRD using LTOT, which can be explained by current knowledge.

## Future Issues

As discussed in this narrative review, cognitive function in patients with CRD using LTOT is associated with many challenges that need to be addressed in the future.

The first challenge lies in understanding the characteristics of cognitive function in patients with CRD using LTOT. In non-LTOT CRD patients, specific cognitive functions, such as verbal memory and executive function, are impaired.^28^,^52^ In addition, COPD reduces dual-tasking ability, and if patients with CRD using LTOT also experience this impairment, it may lead to contact with oxygen delivery equipment and cannula tubing.^53^,^54^ Cognitive functioning in patients with CRD using the LTOT may be specifically impaired, and understanding these characteristics will greatly assist in planning support. For example, a healthcare provider instructing a patient on how to use equipment can provide written instructions for patients with memory impairments or hide unused buttons for patients with attention impairments. Second, we aimed to develop a predictive model for mortality in patients with CRD using the LTOT with added cognitive functions. Prospective cohort studies have identified dyspnea, BMI, exercise capacity, grip strength, and activities of daily living as predictors of mortality in patients with CRD using LTOT.^47^,^55^,^56^ We aim to test a predictive model that integrates these established predictors with cognitive function. This finding provides clues to elucidate how cognitive function interacts with other factors that affect mortality in patients with CRD using LTOT. Third, interventions for the prevention of cognitive impairment in patients with CRD using the LTOT. A report on post-coronavirus disease 2019 (COVID-19) changes showed that even with arterial blood oxygen saturation in the normal range, patients experienced brain hypoxia.^57^ Prevention of cognitive impairment may require setting the oxygen flow rate of the LTOT according to the brain tissue oxygen saturation. Cognitive training may also improve cognitive function for patients with CRD who use LTOT than those who do not use LTOT.^58^,^59^ Additionally, nasal oxygen inhalation has been shown to enhance neural oscillations and improve brain activity and cognitive performance.^60^,^61^ Specifically, this intervention amplifies gamma wave activity within the brain’s default mode network, thereby enhancing higher-order cognitive performance. Interventions that combine oxygen inhalation with cognitive training may offer a noninvasive and effective therapeutic approach for patients with CRD receiving LTOT. Cognitive training combined with exercise may be more effective in improving cognitive function,^62^,^63^ but patients with CRD using LTOT may experience hypoxia during exercise and should be cautious in its application.^63^ Recently, non-invasive brain stimulation has been applied in pulmonary rehabilitation and has the potential to help prevent cognitive impairment in patients with CRD.^64^ In a randomized clinical trial by Andrade et al.^64^ involving intensive care patients with coronavirus disease, the group receiving high-definition transcranial direct current stimulation experienced a shorter duration of delirium compared to the group receiving standard tDCS. The cognitive benefits of tDCS are attributed to its regulation of regional cerebral perfusion and the cortical excitatory effects induced by anodal stimulation.^65^,^66^ Cognitive training that does not require exercise and utilizes modern therapies such as noninvasive brain stimulation may benefit patients with CRD using LTOT. Cognitive training combined with either noninvasive brain stimulation or with nasal oxygen inhalation can target a wide range of cognitive domains and may enhance functions such as memory and attention. Finally, interventions need to be developed to enable patients with CRD and cognitive impairment to use LTOT appropriately. For example, for patients who have difficulty operating oxygen supply equipment, providing guidance to family members may be beneficial.^22^,^67^ Additionally, long-term post discharge support can be provided by incorporating a follow-up program for LTOT through home visits.^68^ Regular follow-ups after LTOT initiation may be an effective intervention for patients with CRD using LTOT and progressive cognitive decline. An educational program for LTOT in patients with cognitive impairment is expected to be developed and validated in the future.

**Figure 2:**
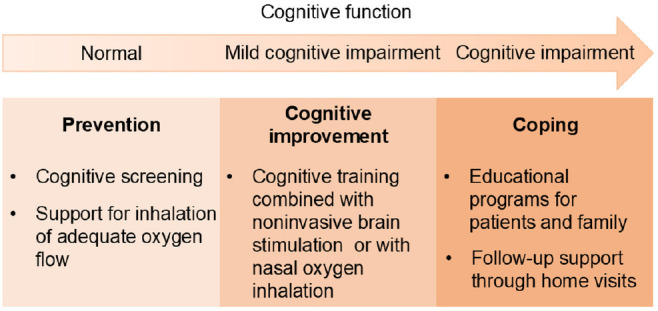
Intervention strategies based on cognitive function in patients with chronic respiratory disease using long-term oxygen therapy. Prevention strategies are recommended for patients with normal cognitive function; cognitive improvement approaches are suitable for those with mild cognitive impairment; and coping strategies are intended for patients with more advanced cognitive impairment. Created with Microsoft PowerPoint.

These strategies can be categorized into three domains: prevention, cognitive improvement, and coping (**Figure 2**). Prevention involves early cognitive screening and the provision of adequate oxygen flow support to reduce the risk of cerebral hypoxia. Such interventions aim to prevent the onset of cognitive impairment. Cognitive interventions enhance cognitive function through training combined with noninvasive brain stimulation and nasal oxygen inhalation. Coping strategies include educational programs for patients and their families, along with follow-up support to ensure sustained adherence to LTOT. These interventions aim to ensure that patients with cognitive impairment consistently use LTOT as prescribed. The selection of prevention, cognitive improvement, and coping strategies should be tailored to the patient’s level of cognitive function.

## Conclusion

This narrative review presents the current knowledge on cognitive impairment in patients with CRD using LTOT. Cognitive function in patients with CRD using LTOT is better preserved than that in patients not using LTOT with comparable respiratory function or hypoxemia but may be worse than that in patients with mildly impaired respiratory function. Cognitive impairment may make it difficult for patients to operate LTOT equipment, which is their lifeline, resulting in a poor prognosis for this patient population.

However, several challenges remain in this research area. In particular, there is an urgent need to investigate strategies to prevent the progression of cognitive decline in patients with CRD using LTOT and develop supportive strategies for this patient population with impaired cognitive function. A more definitive understanding of the relationship between LTOT and cognitive impairment in patients with CRDs would be an important step in addressing this issue.

## Data Availability

*All relevant data are within the paper*.
